# Human Metapneumovirus in Turkey Poults

**DOI:** 10.3201/eid1212.060450

**Published:** 2006-12

**Authors:** Binu T. Velayudhan, Kakambi V. Nagaraja, Anil J. Thachil, Daniel P. Shaw, Gregory C. Gray, David A. Halvorson

**Affiliations:** * University of Minnesota, Saint Paul, Minnesota, USA;; † University of Missouri-Columbia, Columbia, Missouri, USA;; ‡ University of Iowa, Iowa City, Iowa, USA

**Keywords:** human metapneumovirus, turkey, infection, clinical signs, research

## Abstract

This study was conducted to reexamine the hypothesis that human metapneumovirus (hMPV) will not infect turkeys. Six groups of 2-week-old turkeys (20 per group) were inoculated oculonasally with 1 of the following: noninfected cell suspension; hMPV genotype A1, A2, B1, or B2; or avian metapneumovirus (aMPV) subtype C. Poults inoculated with hMPV showed nasal discharge days 4–9 postexposure. Specific viral RNA and antigen were detected by reverse-transcription PCR and immunohistochemical evaluation, respectively, in nasal turbinates of birds exposed to hMPV. Nasal turbinates of hMPV-infected turkeys showed inflammatory changes and mucus accumulation. Each of the 4 hMPV genotypes caused a transient infection in turkeys as evidenced by clinical signs, detection of hMPV in turbinates, and histopathologic examination. Detailed investigation of cross-species pathogenicity of hMPV and aMPV and its importance for human and animal health is needed.

## Abstract 2

TOC summary: Human metapneumovirus causes clinical signs in turkey poults.

Human metapneumovirus (hMPV) is a negative-sense, single-stranded, nonsegmented RNA virus of the genus Metapneumovirus of subfamily Pneumovirinae of the family Paramyxoviridae, a large family of viruses that affect humans and animals (*1*). Recently identified, hMPV is noted for causing respiratory tract infections in children (*2*). The virus was isolated from nasopharyngeal swabs collected from 28 children over a period of 20 years in the Netherlands, and it is thought to have been circulating in human populations for at least 50 years (*2*). hMPV has 2 major genetic lineages (A and B) and at least 4 subgroups identified so far, according to analysis of fusion, attachment glycoprotein, and phosphoprotein genes (*3*). The virus has worldwide distribution, and reports have identified hMPV from the United States, Canada, United Kingdom, Italy, Germany, France, Israel, Australia, Asian countries, and Peru (*4**,**5*).

The disease caused by hMPV ranges from mild upper respiratory tract infection to severe bronchiolitis or bronchitis and pneumonia. It affects all age groups, but it is more severe in young, elderly, and immunocompromised persons (*6*). Serologic surveys indicate that the virus is ubiquitous in nature and that new infections can occur throughout life because of incomplete protection and genetic heterogeneity of the virus (*3**,**7**,**8*). Clinically, the disease is similar to that of human respiratory syncytial virus and is second only to human respiratory syncytial virus as 1 of the leading causes of bronchiolitis in young children (*9*).

Recent studies have shown that avian metapneumovirus (aMPV) subtype C isolates from domestic turkeys and wild birds in the United States show high sequence homology to hMPV (*10**,**11*). Both viruses belong to genus Metapneumovirus and share a projected amino acid identity of 56%–88% (*11*). An upper respiratory tract pathogen of poultry, aMPV has global distribution (*12*). The United States was considered free of aMPV until an outbreak of respiratory tract infection occurred in turkey flocks in Colorado in May 1996 (*13*). Later, in 1997, the disease appeared in Minnesota, the largest turkey-producing state in the country. Serologic evidence now exists for the spread of aMPV into neighboring states of Iowa, Wisconsin, South Dakota, and North Dakota (*14*). The aMPV C identified in the United States has been reported only in North America and is antigenically and genetically different from subtypes A, B, and D detected in Europe, Asia, Africa, and South America (*15**–**18*).

Although hMPV and aMPV are closely related, they are not reported to cause cross-infection. A previous attempt to experimentally infect chickens and turkeys with hMPV was not successful (*2*). Our objective was to reexamine the hypothesis that hMPV will not infect turkeys by exposing 2-week-old turkeys to 4 genotypes of hMPV.

## Materials and Methods

### Cells and Virus

LLC-MK2 cells (ATCC no. CCL-7) were maintained in Minimum Essential Medium (MEM) (Invitrogen, Grand Island, New York, USA) supplemented with 3% bovine fetal serum, 2 mmol/L L-glutamine, nonessential amino acids, 100 IU/mL penicillin G sodium, and 100 μg/mL streptomycin sulfate. Four genotypes of hMPV based on G gene were provided by the University of Iowa for this study: A1 (GenBank accession no. DQ312456), A2 (DQ312449), B1 (DQ312452), and B2 (DQ312457). Genotype A1 was isolated from a nasal wash sample of a 3-year-old girl by propagation on LLC-MK2 cells for 29 days. Genotypes A2 and B1 were isolated from nasal wash samples of 7- and 1-year-old girls, respectively, by propagation on LLC-MK2 cells for 14 days. Genotype B2 was isolated from a nasopharyngeal sample of a 19-year-old woman by propagation on LLC-MK2 cells for 20 days. After primary isolation, these viruses were further propagated in LLC-MK2 cells in opti-MEM (Invitrogen) with 2 μg/mL trypsin (replenished every other day), 100 IU/mL of penicillin G sodium, 100 μg/mL streptomycin, and no serum.

aMPV C was isolated from the nasal turbinates of 8-week-old turkeys with acute upper respiratory tract infection. The virus was passaged 6 times on chicken embryo fibroblasts, then 6 times on Vero cells. The virus, designated as aMPV/Minnesota/Turkey/19/2003 (aMPV MN 19), was used with a titer of 10^5^ 50% tissue culture infective dose (TCID_50_)/mL.

### Birds

A total of 120 2-week-old turkey poults (Large White, Nicholas) from an aMPV-naive breeder flock that was not vaccinated for aMPV were used (Institutional Biosafety Committee [ID 785] and Institutional Animal Care and Use Committee approved protocol #0505A69986, University of Minnesota, Saint Paul, MN, USA). These turkey poults were free of aMPV, Mycoplasma meleagridis, M. synoviae, M. gallisepticum, Bordetella avium, and Newcastle disease virus.

### Experimental Design

Turkey poults were divided into 6 groups of 20 each. Poults in each group were inoculated oculonasally with 200 μL (50 μL each in each eye and nostril) of 1 of the following: noninfected LLC-MK2 cell suspension (sham-inoculated controls), 1 of the 4 genotypes of hMPV (A1, A2, B1, B2), or aMPV C (positive controls). Poults in the 4 treatment groups received fresh, untitrated virus propagated in LLC-MK2 cells. To ascertain the amount of virus inoculated into poults in each group, each inoculum was later titrated by serial dilutions in LLC-MK2 cells. Poults were monitored daily for clinical signs. Two randomly selected poults from each group were killed for necropsy and sample collection at days 3, 5, and 7 postexposure. Nasal turbinates, tracheas, and lungs were tested for viral RNA by reverse-transcription (RT) PCR with specific primers (consensus primers for all of the 4 genotypes) for hMPV and aMPV as described below. Tissue sections were stained with hematoxylin and eosin and examined for histopathologic lesions and for viral antigen by immunohistochemical methods (*19*). Sera collected from poults in each group at days 14 and 21 postexposure were tested with aMPV-ELISA (*20*). No convincing evidence exists for any cross-reactivity between hMPV and aMPV.

### Clinical Sign Scoring

Turkey poults were monitored daily for any clinical signs. A clinical sign scoring system was used to record signs shown by those exposed to the viruses (*21*). Briefly, poults in each group were given a score of 0 when they showed no clinical signs; a score of 1 for unilateral nasal discharge; 2 for bilateral nasal discharge; or 3 for thick, copious, bilateral nasal discharge. Unilateral sinus swelling was recorded as score 1, bilateral sinus swelling as 2, unilateral conjunctivitis as 1, and bilateral conjunctivitis as 2. The total score for each bird was expressed as the sum of individual scores mentioned above.

### RT-PCR

Nasal turbinates, tracheas, and lungs were collected in MEM (Invitrogen) containing 100 IU/mL penicillin G sodium and 100 μg/mL streptomycin to prepare a 20% tissue homogenate. After centrifugation at 3,000 × g for 10 min to remove tissue debris, an additional centrifugation was performed at 8,000 × g for 10 min, and the supernatant was collected and used for RT-PCR. Viral RNA was extracted from the tissue homogenate supernatant by using a commercial viral RNA extraction kit (Qiagen, Valencia, CA, USA). One-step RT-PCR was performed by using a commercially available 1-step RT-PCR kit (Qiagen). Two primers designed on the basis of the hMPV fusion protein gene, 5´-GAGCAAATCCCAGACA-3´ and 5´-GAAAACTGCCGCACAACATTTAG-3´, were used as forward and reverse primers, respectively (*22*). A 50-μL reaction was set for each tissue sample with the following temperature conditions: reverse transcription at 50°C for 30 min, initial denaturation at 94°C for 10 min, 35 cycles of annealing at 54°C for 1 min, extension at 72°C for 1 min, and denaturation at 94°C for 30 sec, followed by a final extension at 72°C for 10 min. The RT-PCR products were then analyzed by electrophoresis on a 1.2% agarose gel. A separate RT-PCR was set for each tissue sample by using specific primers designed from the matrix protein gene of aMPV to look for any cross-reactivity. The protocol described by Shin et al. (*23*) was used. The forward primer was 5´-ACAGTGTGTGAGTTAAAAG-3´ (M1) starting from base 335, and the reverse primer was 5´- TGACTTCAGGACATATCTC-3´ (M2) starting from base 754 of the US isolate of aMPV (aMPV/Minnesota/Turkey/2a/1997).

### Virus Isolation

Nasal turbinate homogenate from poults exposed to the 4 genotypes of hMPV was injected onto LLC-MK2 cells for virus isolation. Five blind passages (14 days each) were done, and the cells were examined for any cytopathic effects. At the endpoint of each passage, 3 cycles of freeze-thaw were done and the cell culture supernatant was tested for hMPV RNA by RT-PCR as described above.

### Histopathologic and Immunohistochemical Evaluations

Nasal turbinates, tracheas, and lungs were fixed in 10% buffered neutral formalin. Tissue sections were stained with hematoxylin and eosin and examined for histopathologic changes.

An immunoperoxidase procedure (*19*) originally developed to detect aMPV antigen was modified to detect hMPV antigen in formalin-fixed nasal turbinate, trachea, and lung tissues by using hMPV B2 polyclonal sera from rabbits. The tissue sections were also tested by using aMPV polyclonal sera from rabbits (*19*).

### Serologic and Bacteriologic Examination

Serum samples were collected from turkey poults at days 14 and 21 postexposure. Samples were examined for antibodies by an aMPV-ELISA (*20*) that used aMPV subtype C whole-antigen–coated plates and anti-turkey IgG conjugate as the secondary antibody.

To exclude the possibility of any bacterial infections, nasal and tracheal swabs were streaked on blood agar and McConkey agar plates. The plates were incubated at 37°C for 3 days, after which they were examined for any bacterial pathogens.

## Results

### Virus Titers

The 4 genotypes of hMPV showed cytopathic effects in LLC-MK2 cells 10–14 days postinoculation. Cytopathic effects consisted mainly of cell rounding and formation of syncytium. hMPV genotypes A1, A2, and B1 inocula had a titer of 10^3^ TCID_50_/mL each. Genotype B2 inoculum had a titer of 10^5^ TCID_50_/mL. The aMPV C inoculum had a titer of 10^5^ TCID_50_/mL.

### Clinical Sign Scoring

**Figure 1:**
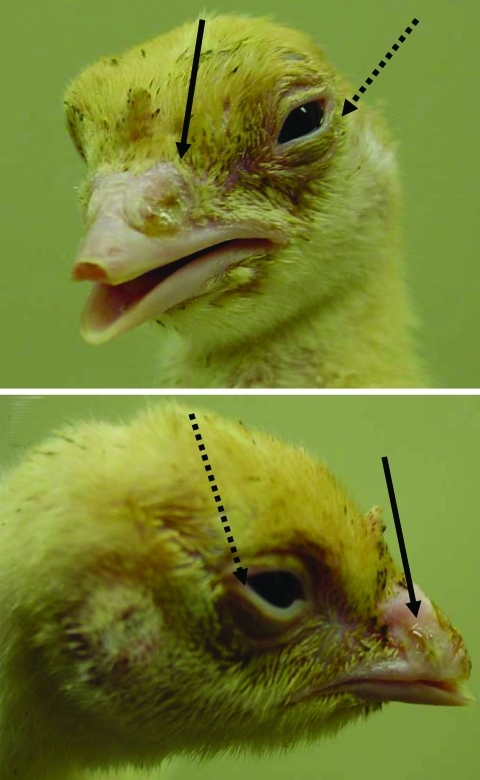
Clinical signs of turkey poults exposed to human metapneumovirus B2, showing nasal discharge (solid arrows) and swollen eyelids (dotted arrows).

Poults inoculated with any of the 4 hMPV genotypes had unilateral or bilateral nasal discharge (Table 1; Figure 1), which varied from watery to thick mucus. Clinical signs started on day 4 postexposure and stopped on day 9 postexposure. Those infected with hMPV A1 had a clinical score range of 1–2, and 6 of 20 showed clinical signs. Poults infected with hMPV A2 had a score range of 1–8, and 12 of 20 showed clinical signs. Those inoculated with hMPV B1 had a score range of 1–7, and 7 of 20 showed clinical signs. The group inoculated with hMPV B2 had the most poults that showed clinical signs (14 of 20); score range was 2–8. Poults inoculated with aMPV MN 19 showed severe clinical signs and had an average score of 14.17 (Table 1). The main signs were thick, mucous, bilateral nasal discharge and infraorbital sinus swelling.

**Table 1:** Clinical sign scores of turkey poults exposed and not exposed to human or avian metapneumovirus*

Treatment group	Clinical sign score						Average score
	4 d (n = 18)	5 d (n = 18)	6 d (n = 16)†	7 d (n = 16)†	8 d (n = 14)‡	9 d (n = 14)‡	
Noninfected control	0	0	0	0	0	0	0
hMPV A1	2.0	2.0	2.0	2.0	1.0	0	1.5
hMPV A2	8.0	6.0	4.0	4.0	3.0	1.0	4.34
hMPV B1	1.0	1.0	4.0	7.0	3.0	0	2.66
hMPV B2	8.0	6.0	8.0	8.0	6.0	2.0	6.34
aMPV C	14.0	15.0	15.5	16.5	13.5	10.5	14.17

*d, days postexposure; hMPV, human metapneumovirus genotypes A1, A2, B1, B2; aMPV C, avian metapneumovirus subtype C. †For the group exposed to hMPV B2, n = 15 because of 1 death (cause unknown) on day 5 postexposure. ‡For the group exposed to hMPV B2, n = 13 because of 1 death (cause unknown) on day 5 postexposure.

### RT-PCR

At day 3 postexposure, RT-PCR detected hMPV viral RNA in the nasal turbinates of poults in each group exposed to hMPV. Viral RNA was also detected at day 5 postexposure in 1 bird exposed to hMPV B2 (Table 2). Tracheas and lungs did not show any viral RNA. Sham-inoculated birds were negative for hMPV. The aMPV RNA was not detected in unexposed or hMPV-exposed poults. Those infected with aMPV showed viral RNA in nasal turbinate (Table 2) and trachea homogenate (data not shown) by aMPV specific RT-PCR on days 3, 5, and 7 postexposure.

**Table 2:** Reverse-transcription PCR detection of hMPV in nasal turbinates of turkey poults exposed and not exposed to human or avian metapneumovirus*

Treatment group	No. with hMPV viral RNA		
	3 d (n = 2)	5 d (n = 2)	7 d (n = 2)
Noninfected control	0	0	0
hMPV A1	1	0	0
hMPV A2	1	0	0
hMPV B1	1	0	0
hMPV B2	2	1	0
aMPV C	2	2	2

*hMPV, human metapneumovirus genotypes A1, A2, B1, B2; d, days postexposure; aMPV C, avian metapneumovirus subtype C.

### Virus Isolation

No cytopathic effects were detected in LLC-MK2 cell cultures inoculated with the nasal turbinate homogenate from birds exposed to any one of the 4 genotypes of hMPV. No hMPV RNA was detected from the cell culture supernatant by RT-PCR after 5 blind passages.

### Histopathologic and Immunohistochemical Evaluations

**Figure 2:**
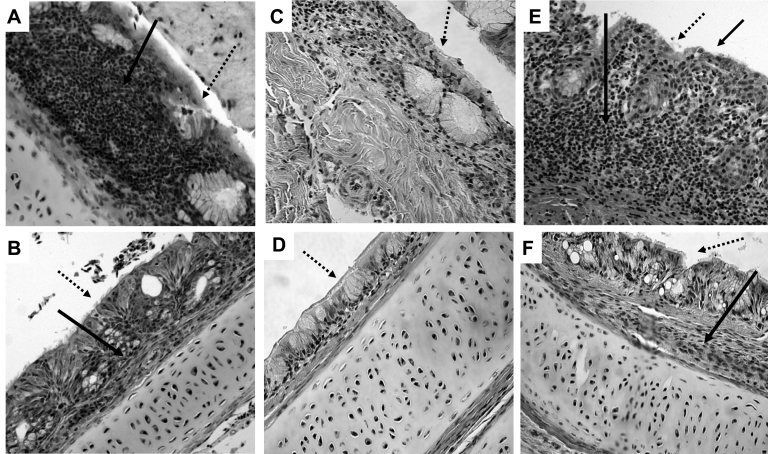
Histopathologic appearance of nasal turbinate and trachea tissue (magnification ×200). A) Nasal turbinates of turkey poults exposed to human metapneumovirus (hMPV) B2, showing infiltration of inflammatory cells (hematoxylin and eosin staining; solid arrow ). B) Trachea of turkey poults exposed to hMPV B2, showing mild inflammation with infiltration of a few inflammatory cells in the lamina propria (solid arrow). C) Nasal turbinate of sham-inoculated turkey poults. D) Trachea of sham-inoculated turkey poults. E) Nasal turbinate of turkey poults exposed to avian metapneumovirus (aMPV C), showing infiltration of inflammatory cells and multifocal loss of cilia (solid arrows). F) Trachea of turkey poults exposed to aMPV C, showing mild inflammation with infiltration of a few inflammatory cells in the lamina propria (solid arrow). Dotted arrows indicate mucosal surface.

At days 3 and 5 postexposure, each of the 4 genotypes had caused mild to moderate histopathologic lesions in the nasal turbinates of poults inoculated with hMPV. Inflammatory changes were more pronounced in those inoculated with hMPV B2 than with any of the other 3 genotypes. Lesions consisted of infiltration of inflammatory cells in the lamina propria, mainly lymphocytes, macrophages, and plasma cells (Figure 2A). Mucus had accumulated in the nasal cavity, and mucous glands had dilated. Tracheas of poults inoculated with hMPV B2 showed mild inflammatory changes in the form of infiltration of inflammatory cells on day 5 postexposure (Figure 2B), whereas lungs did not show any histopathologic lesions. Tissues from sham-inoculated poults also did not show any lesions (Figure 2, C and D). Nasal turbinates and tracheas of poults infected with aMPV showed infiltration of inflammatory cells (Figure 2, E and F), dilation of mucosal glands, and multifocal loss of cilia in turbinates (Figure 2E).

**Figure 3:**
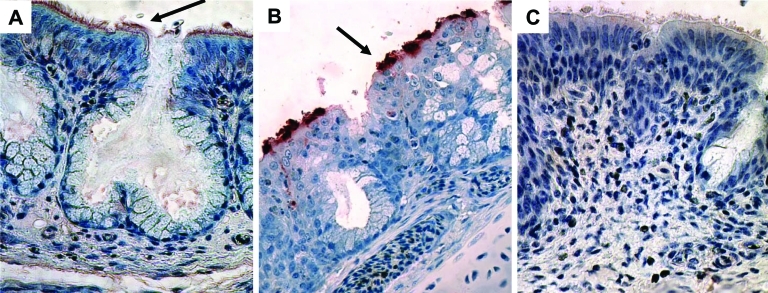
Immunohistochemical findings for nasal turbinates, showing peroxidase staining of viral antigen in the epithelial surface (magnification ×400; arrows). A) Turkey poults exposed to human metapneumovirus (hMPV B2). B) Turkey poults exposed to avian metapneumovirus (aMPV C). C) Sham-inoculated turkey poults.

On day 3 postexposure, immunohistochemical evaluation showed hMPV antigen in the epithelial surface of nasal turbinates of poults in each group inoculated with hMPV (Figure 3A). No antigen was detected in tracheas or lungs. On days 3, 5, and 7 postexposure, those infected with aMPV showed viral antigen in turbinate (Figure 3B) and trachea tissues (data not shown). Sham-inoculated birds did not show antigen in tissues (Figure 3C).

### Serologic and Bacteriologic Examination

No antibodies were detected by aMPV-ELISA in sera collected from poults exposed to hMPV at 14 and 21 days postexposure. However, aMPV-infected poults had positive aMPV-ELISA results at 14 and 21 days postexposure (data not shown). No cross-reactivity between aMPV and hMPV was detected by either test conducted using aMPV specific reagents, whereas their respective controls with aMPV-infected samples showed positive reactions. No pathogenic bacterial colonies were isolated from nasal or tracheal swabs collected from any of the 120 poults.

## Discussion

All 4 genotypes of hMPV did infect turkeys, as evidenced by clinical signs, RT-PCR results, immunohistochemical findings, and histopathologic changes in the nasal turbinates and tracheas of exposed turkeys. These findings contradict those of van den Hoogen et al., who detected neither clinical signs nor virus replication in juvenile turkeys inoculated with hMPV nor experimental reproduction of infection in chickens (*2*).

In our study, 2-week-old turkeys in the four groups were inoculated oculonasally with hMPV. Those receiving either dose of hMPV (2×10^2^ TCID_50_ or 2×10^4^ TCID_50)_ developed clinical signs. In contrast, van den Hoogen et al. inoculated 5×10^4^ TCID_50_ of hMPV in the conjunctivae and respiratory tracts of 4 juvenile turkeys (age and breed of turkeys were not mentioned) and over a 3-week period examined the birds for clinical signs and virus replication by sampling cloacal and throat swabs. Although they inoculated more virus than we did, their birds did not show clinical signs, so the amount of virus inoculated may not be the reason for the different results. Possible reasons for the contradictory results could be origin of the virus; source, age, and immune status of the turkeys used; and the samples that were analyzed. With aMPV infection, younger turkeys show more severe clinical signs than older turkeys (*19**,**21*); and nasal turbinate, a major predilection site for aMPV replication in turkeys (*21*), is where we detected hMPV.

In our study, severity of clinical signs and lesions varied with different genotypes, possibly because of differences in titers inoculated, which can potentially influence virus dissemination and pathogenicity. The virus inocula were titrated in LLC-MK2 cells after inoculating birds. The endpoint of titration was 14 days postinoculation of the virus in cell cultures; to ensure fresh inocula, we decided to use untitrated virus. The main limitation of this approach was that we could not use a uniform titer of each virus inoculum. Therefore, we could not infer any information about differences in severity of infection in turkeys with respect to different genotypes of hMPV.

Clinical signs appeared in 30%–70% birds in the groups exposed to the 4 genotypes of hMPV. The clinical signs were similar to those of birds experimentally infected with aMPV C (*21*). The main clinical sign observed in birds infected with aMPV C is watery to thick mucous discharge (*21*), and our poults exposed to hMPV showed watery to thick mucous nasal discharge. The main difference in clinical signs in our poults infected with hMPV was the absence of infraorbital sinus swelling, which is often associated with aMPV infection in turkeys (*21*).

Infiltration of inflammatory cells was observed in the nasal turbinates of poults infected with hMPV and aMPV. Nasal turbinates of poults infected with aMPV showed multifocal loss of cilia, whereas this lesion was not observed in those infected with hMPV. Immunohistochemical evaluation showed hMPV antigen in the epithelial surface of nasal turbinates; however, the staining was not as intense as we observed from aMPV infections (Figure 3B). The lack of detection of hMPV antibodies in the sera and antigens in tissues by serologic testing and immunohistochemical evaluation using aMPV reagents, respectively, indicates little to no cross-reactivity between aMPV and hMPV. However, cross-reactivity between a conserved region in aMPV nucleoprotein (N) with hMPV has been reported (*24*). We think that whole aMPV virus antigen or aMPV polyclonal serum-based detection systems may not work in the same way as affinity-purified anti-N or monoclonal antibodies.

A synthetic aMPV N-peptide–based ELISA could detect aMPV subtypes A, B, and C (*25*), whereas whole-virus–based ELISAs with plates coated with European subtypes of aMPV antigen could not detect aMPV C antibodies and vice versa (*25*). Even with aMPV subtypes, the whole-virus antigen-based detection systems did not show cross-reaction between subtypes, whereas a conserved region of nucleocapsid protein or synthetic peptide–based detection systems showed cross-reactivity. This could also occur with cross-reaction between hMPV and aMPV.

Possible zoonotic potential of metapneumoviruses and their coexistence, if any, across species barriers must be considered. Recent studies have shown that most infectious agents, especially newly emerging pathogens, can be transmitted between humans and animals (*26*). Taylor et al. reviewed literature on 1,415 species of human pathogens and identified 61% of them and 75% of emerging human disease pathogens as zoonotic. In this context, our data suggest hMPV and aMPV merit further investigations regarding cross-species transmission (*26*).

Mice, cotton rats, hamsters, ferrets, and primate species such as rhesus monkeys and African green monkeys have been used to experimentally reproduce the disease caused by hMPV (*27**,**28*). Hamsters, ferrets, and green monkeys have been shown to replicate hMPV and produce neutralizing antibodies (*27*). Replication of the virus in infected lung was also experimentally shown in mice and cotton rats (*28*). Although hMPV replicated in the upper and lower respiratory tract of hamsters and ferrets, these animals did not show any clinical signs (*27*). Hamelin et al. reported that BALB/c mice infected with hMPV showed ruffled hair, breathing difficulty, and weight loss (28). On the other hand, our infected turkeys showed respiratory signs in the form of watery to thick mucous nasal discharge and eyelid swelling.

Histopathologic changes in the form of interstitial inflammation and alveolitis have been observed in cotton rats and mice infected with hMPV (*28*). In our turkey poults, we did not detect inflammatory lesions in the lungs, although nasal turbinates and tracheas showed infiltration of inflammatory cells.

In our study, each of the 4 genotypes of hMPV caused a transient infection in turkey poults, as evidenced by clinical sign scores from days 4 to 9 postexposure, detection of hMPV RNA in nasal turbinates at days 3 and 5 postexposure, and histopathologic lesions in the turbinates and tracheas. Ours is the first report of an experimental infection of turkeys with hMPV, and it opens up the possibility of using turkeys as infection models for this virus. Our data indicate a need for detailed investigation of the cross-species pathogenicity of hMPV and aMPV and significance of these viruses for human and animal health.
